# Expression, purification, and characterization of the recombinant exo-1,3-β-glucanase (Exo1) of the pathogenic oomycete *Pythium insidiosum*

**DOI:** 10.1016/j.heliyon.2020.e04237

**Published:** 2020-06-19

**Authors:** Tiwa Rotchanapreeda, Yothin Kumsang, Pattarana Sae-Chew, Thidarat Rujirawat, Tassanee Lohnoo, Wanta Yingyong, Penpan Payattikul, Onrapak Reamtong, Theerapong Krajaejun

**Affiliations:** a Department of Pathology, Faculty of Medicine, Ramathibodi Hospital, Mahidol University, Bangkok, Thailand; b Research Center, Faculty of Medicine, Ramathibodi Hospital, Mahidol University, Bangkok, Thailand; c Department of Molecular Tropical Medicine and Genetics, Faculty of Tropical Medicine, Mahidol University, Bangkok, Thailand

**Keywords:** Microbiology, Mycology, Protein engineering, Medical microbiology, Vaccines, *Pythium insidiosum*, Pythiosis, Exo-1,3-β-glucanase, Exo1, Recombinant protein, Immunoreactive protein

## Abstract

Pythiosis is a deadly infectious disease of humans and animals living in tropical and subtropical countries. The causative agent is the oomycete *Pythium insidiosum*. Treatment of pythiosis is challenging. The use of antimicrobial agents usually fails in the treatment of pythiosis. Many patients undergo surgical removal of an infected organ (i.e., eye, arm, and leg). The immunotherapeutic vaccine, prepared from the crude extract of *P. insidiosum*, shows limited efficacy against pythiosis. The fatal outcome occurs in patients with advanced disease. There are urgent needs for an effective therapeutic modality for pythiosis. Recently, the exo-1,3-β-glucanase (Exo1) has been identified as a conserve immunoreactive protein of *P. insidiosum*. Exo1 was predicted to reside at the cell membrane and hydrolyze cell wall β-glucan during cell growth. An Exo1 ortholog is absent in the human genome, making it an appealing target for drug or vaccine development. We attempted to clone and express the codon-optimized *exo1* gene of *P. insidiosum* in *E. coli*. To solve the inclusion body formation, expression and purification of Exo1 were achievable in the denaturing condition using SDS- and urea-based buffers. Exo1 lacked hydrolytic activity due to the absence of proper protein folding and post-translational modifications. ELISA and Western blot analyses demonstrated the immunoreactivity of Exo1 against pythiosis sera. In conclusion, we successfully expressed and purified the immunoreactive Exo1 protein of *P. insidiosum*. The recombinant Exo1 can be produced at an unlimited amount and could serve as an extra protein to enhance the effectiveness of the current form of the vaccine against pythiosis.

## 1 Introduction

Oomycetes are aquatic, filamentous, eukaryotic microorganisms that are phylogenetically and biochemically distinct from fungi (Lévesque, 2011; Gaastra et al., 2010). Most pathogenic oomycetes cause an infection in plants, while just a few species can infect animals (Kamoun, 2003). *Pythium insidiosum* is a prominent oomycete that can infect humans and animals and causes the deadly tropical disease called “pythiosis” (De Cock et al., 1987; Gaastra et al., 2010; Kamoun, 2003). The treatment of pythiosis is challenging. The use of antimicrobial agents usually fails in the treatment of pythiosis. Many patients undergo surgical removal of an infected organ (i.e., eye, arm, and leg) to limit the disease progression (Krajaejun et al., 2006a; Gaastra et al., 2010). The immunotherapeutic vaccine, prepared from the crude protein extract of *P. insidiosum*, shows a limited efficacy against pythiosis (Krajaejun et al., 2006a). The fatal outcome occurs in patients with advanced disease. There are urgent needs for an effective therapeutic modality for pythiosis.

Little is known about the biology and pathogenesis of *P. insidiosum* at cellular and molecular levels. The *exo1* gene (2,229 bases long) encoding a putative exo-1,3-β-glucanase (Exo1) has been identified in *P. insidiosum* (Krajaejun et al. 2006b, 2010). Exo1 is an immunoreactive protein that upregulates at body temperature and triggers host antibody response in pythiosis patients (Krajaejun et al. 2006b, 2010; Keeratijarut et al., 2015). Unlike fungi, the cell wall of oomycetes primarily composes of cellulose, glucans, and to a lesser extent, chitin (Mélida et al., 2013). β-glucan is a primary carbohydrate found in the *P. insidiosum* extract (Tondolo et al., 2017). The β-glucanase Exo1 may involve in cell wall remodeling, hyphal growth, and invasion of *P. insidiosum* (Keeratijarut et al., 2015). The Exo1-coding sequence is absent from the human genome, making it an appealing diagnostic and therapeutic target of *P. insidiosum* (Keeratijarut et al., 2015).

Expression and purification of Exo1 from the bacterium *E. coli* have failed, possibly due to the recombinant Exo1 is either water-insoluble, toxic, unstable, or degraded (Keeratijarut et al., 2015). The current study attempted to clone and express the synthetic codon-optimized *exo1* gene of *P. insidiosum* in a selected *E. coli* strain. The soluble recombinant Exo1 protein was successfully generated and purified, and it could serve as a candidate for downstream applications, such as the development of an effective vaccine against pythiosis.

## 2 Materials and methods

### 2.1 Ethical consideration

This work was approved by the Committee for Research, Faculty of Medicine Ramathibodi Hospital, Mahidol University (Approval number: MURA2020/122).

### 2.2 Bioinformatic analyses of Exo1

Exo1 homologous protein sequences of *P. insidiosum* (GenBank accession: BAS04780.1), several other oomycetes [i.e., *Pythium ultimum* (FungiDB accession: PYU1_T011822), *Saprolegnia parasitica* (FungiDB accession: SPRG_13455), *Hyaloperonospora arabidopsidis* (FungiDB accession: HpaG806582), *Phytophthora infestans* (GenBank accession: XP_002903391.1), *Phytophthora parasitica* (FungiDB accession: PPTG_01939), and *Phytophthora sojae* (GenBank accession: XP_009531404.1)] and fungi [i.e., *Aspergillus fumigatus* (GenBank accession: XP_750110.1), *Candida albicans* (GenBank accession: CAA39908.1), and *Saccharomyces cerevisiae* (GenBank accession: AAB24895.1)] were retrieved from the GenBank (https://www.ncbi.nlm.nih.gov/) and FungiDB (Basenko et al., 2018) databases. Protein architectures (i.e., lengths and domain organizations) of these Exo1 homologs were predicted using the InterPro program (https://www.ebi.ac.uk/interpro/) and depicted using the Illustrator for Biological Sequences program (http://ibs.biocuckoo.org/). The biochemical properties of Exo1 were predicted using the ScanProsite program (https://prosite.expasy.org/scanprosite/) and ProtParam program (https://web.expasy.org/protparam/).

### 2.3 Plasmid DNA construction

**Figure 1:**
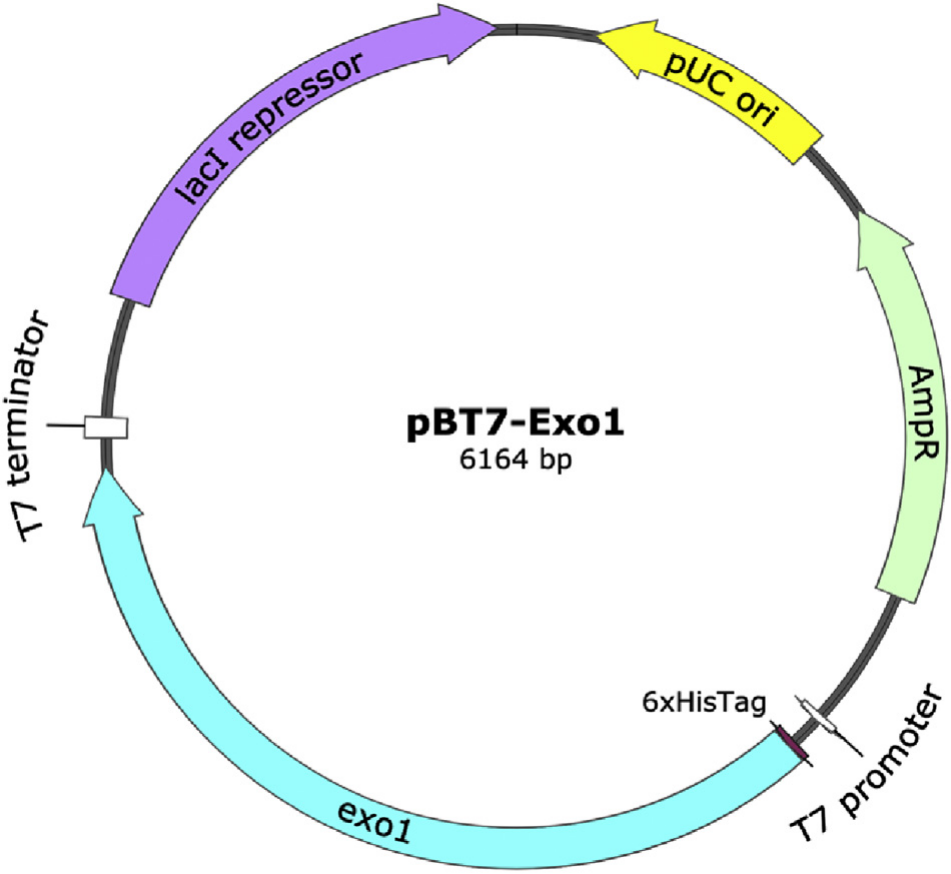
The plasmid pBT7-Exo1 (6,164 bp in size) used for expressing the recombinant Exo1 protein of *P. insidiosum*. The plasmid contains a nearly-complete, signal peptide-excluded, codon-optimized *exo1* coding sequence. The components required for protein expression [i.e., T7 promoter and terminator, *exo1*-coding sequence (2,163 bp long), histidine tag (6xHisTag), selection marker (AmpR), the origin of replication (pUC ori) and lacI repressor] are shown.

A nearly-complete *exo1*-coding sequence of *P. insidiosum* (2,163 bp long; accession number: LC033486), excluding the signal peptide code, was codon-optimized for *E. coli*, synthesized, and cloned into the expression vector, pBT7-N-His by Bioneer corporation (Bioneer, Korea). The 6xHis tag-coding sequence was added to the Exo1 N-terminus due to the feasibility of removing the tag by exopeptidases when needed (Pedersen et al., 1999). The resulting plasmid DNA, pBT7-Exo1 (Figure 1), was maintained in the *E. coli* strain DH5α.

### 2.4 Expression and purification of the recombinant Exo1

The plasmid pBT7-Exo1 was transformed into the *E. coli* strain BL21(DE3)pLysS. The transformed bacteria were transferred to LB broth (BD Difco) containing 100 μg/ml ampicillin and shaking incubated (200 rpm) at 37 °C overnight. Bacterial growth was adjusted to 0.1 optical density at the 600-nm wavelength (OD_600_) using the ampicillin-containing LB broth and incubated at 37 °C to reach 0.6–0.8 OD_600_. The bacterial culture was induced for protein expression by 0.1 mM IPTG and incubated at 25 °C for 3 h. The uninduced culture (without IPTG) was performed in parallel. A cell pellet was harvested from 100 ml of the bacterial culture by centrifugation (3,000 ×g) at 4 °C for 10 min.

Ten milliliters of SDS lysis buffer (50 mM Tris-HCl pH 8.0, 150 mM NaCl, 10% glycerol, 1% SDS, 1 mM PMSF) was added to the harvested cell pellet. After the bacterial cells were lysed by vigorously vortexing, the obtained crude lysate was centrifuged (6,000 ×g) at 4 °C for 15 min. The supernatant was transferred to a new tube, incubated on ice for 1 h, and centrifuged to precipitate SDS crystalline. The resulting supernatant (~10 ml) was dialyzed against 1,000 ml of dialysis buffer (50 mM Tris-HCl pH 8.0, 150 mM NaCl, 10% glycerol) using a SnakeSkin dialysis tube (22-mm diameter; 10,000 MWCO; Thermo Scientific). The dialysis was performed at room temperature for 4 h. After the buffer was replaced, the dialysis was continued at 4 °C overnight.

The recombinant Exo1 protein was purified using an ÄKTA pure chromatography system machine (GE Healthcare). In brief, the dialyzed protein sample was loaded into a 5-ml-size HisTrap FF Ni sepharose column (GE Healthcare) and equilibrated with the binding buffer (50 mM Tris-HCl pH 8.0, 500 mM NaCl, 8 M urea, 40 mM imidazole). The protein-bound column was washed with 5 column volumes of the binding buffer. A linear gradient of the elution buffer (50 mM Tris-HCl pH 8.0, 500 mM NaCl, 8 M urea, 1 M imidazole) was used to strip the bound recombinant protein from the column. Protein concentration was measured using a NanoDrop 2000 spectrophotometer (Thermo Scientific) with an extinction coefficient of 171,615 M^−1^ cm^−1^. The purified Exo1 was stored at 4 °C.

### 2.5 Validation of the recombinant Exo1 by Western blot and mass spectrometry

The purified Exo1 protein was separated by SDS-PAGE (4% stacking and 12% separating gel) and blotted onto the 0.45-μm-pore-size nitrocellulose membrane (Bio-Rad) using Power Blotter XL semi-dry transfer (Invitrogen). A blotted membrane was incubated with the blocking buffer [5% skim milk in TBS-T (20 mM Tris, 150 mM NaCl, 0.1% Tween 20, pH 7.4)] at 4 °C overnight. After washing 3 times (5 min each) with TBS-T, the blotted membrane was probed with either the mouse anti-6x histidine tag monoclonal antibody (Abcam) or the rabbit anti-Exo1 peptide sera (Keeratijarut et al., 2015) (1:5,000 in blocking buffer) at room temperature for 3 h. After another washing step, the corresponding conjugate [i.e., the goat anti-mouse IgG conjugated with horseradish peroxidase (Bio-Rad) (1:5,000 in blocking buffer) or the goat anti-rabbit IgG conjugated with alkaline phosphatase (Abcam) (1:40,000 in blocking buffer)] was incubated with the membrane at room temperature for 2 h. The membrane was washed 3 times with TBS-T and washed twice with either PBS (10 mM Na_2_HPO_4_, 1.8 mM KH_2_PO_4_, 137 mM NaCl, 2.7 mM KCl, pH 7.4) (for horseradish peroxidase conjugate) or alkaline phosphatase buffer (100 mM Tris, 100 mM NaCl, 5 mM Mg_2_Cl, pH 9.5) (for alkaline phosphatase conjugate). Western blot signals were developed using an appropriate substrate: (i) DAB (0.03%), CoCl_2_ (0.05%), and H_2_O_2_ (0.03%) in PBS (for horseradish peroxidase); or (ii) NBT (0.033%) and BCIP (0.0165%) in alkaline phosphatase buffer (for alkaline phosphatase). Developed membranes were documented using a ChemiDoc MP Imaging System machine (Bio-Rad).

For mass spectrometric analysis, 5 μg of purified Exo1 were separated by SDS-PAGE. The 82-kDa protein was excised from the Coomassie brilliant blue-stained SDS-PAGE gel, digested with trypsin, and subject to LC-MS/MS analysis using an Ultimate 3000 nano-LC system (Dionex, Surrey, UK) coupled with microTOF-Q II (Bruker, Bremen, Germany) (Rujirawat et al., 2018). The LC-MS/MS-derived data were used for peptide mapping against the Mascot library prepared from 14,962 theoretically-trypsin-digested proteins of *P. insidiosum* (Rujirawat et al., 2018).

### 2.6 Refolding of denatured Exo1 protein

Several affinity chromatography-derived fractions containing the denatured Exo1 (solubilized in 8 M urea) were combined, centrifuged using an Amicon Ultra-15 centrifugal filter tube (10,000 MWCO; Merck), and adjusted to 10 mg/ml with the elution buffer (without imidazole). Protein refolding was performed by 2 methods: dilution and step-wise dialysis. The dilution-based method employed a Pierce protein refolding kit (Thermo Scientific). Several Tris-based refolding buffers were mixed with guanidine hydrochloride, L-arginine, EDTA, reduced glutathione, or oxidized glutathione. The denatured Exo1 (50 μl) was added to each refolding buffer (950 μl), vortexed, and incubated at 4 °C for 24 h. For the step-wise dialysis method, 100 μl of the denatured Exo1 was loaded into a Slide-A-Lyzer MINI Dialysis cup (3,500 MWCO; Thermo Scientific) and sequentially dialyzed (at 4 °C for 2 h) against 1,000 ml of the dialysis buffers (5, 3 and 1 M urea in 50 mM Tris-HCl pH 8.0 and 500 mM NaCl). Exo1 was further dialyzed using the protein storage buffer (50 mM Tris-HCl pH 8.0, 150 mM NaCl, 10% glycerol) to remove urea.

The native PAGE was used to check protein refolding and solubility. The denatured Exo1 was mixed with the protein loading dye (containing SDS and DTT) and boiled. Each protein refolding product was mixed with the Purple Gel Loading Dye no SDS (NEB), and loaded into a continuous PAGE gel comprising 10% polyacrylamide, 375 mM Tris-HCl (pH 8.8), 0.1% APS, and 0.1% TEMED. Protein electrophoresis was carried out using the Tris-glycine running buffer (25 mM Tris, 192 mM glycine) at 100 V for 120 min. The native PAGE gel was stained with Coomassie brilliant blue.

### 2.7 Biochemical analyses of the refolding Exo1

Protein refolding products (from the dilution and step-wise dialysis methods) were tested for hydrolytic activity against the cellulose Avicel PH-101 (Sigma) and the polysaccharide laminarin (Sigma). An agar plate was prepared from 1.5% Technical agar (BD Difco) in 15 ml of TBS (20 mM Tris, 150 mM NaCl, pH 7.4) or sodium acetate buffer (70 mM CH_3_COONa, 30 mM CH_3_COOH, pH 5.0), in the presence of 1.5% Avicel (mixed in the agar) or 150 μl of 1% laminarin (overlaid on the agar). A stack of 3 Whatman antibiotic assay discs (6-mm in diameter; GE Healthcare) was saturated with 90 μl of a refolding protein and placed on an agar plate. A disc soaked with 30 μl of 100 mg/ml (in sodium acetate buffer) of either *Trichoderma reesei*'s cellulase (Sigma) or *Trichoderma harzianum*'s lysing enzymes (Sigma) served as a positive control, whereas 3 discs soaked with 90 μl of Tris-based refolding buffer served as a negative control. The plates were incubated at 37 °C for 18 h, stained with Lugol's iodine solution (0.33% I_2_, 0.66% KI) for 5 min, and de-stained with water to observe a hydrolytic (clear) zone.

The 3,5-dinitrosalicylic acid (DNS) assay (Miller, 1959) was also used to determine the hydrolytic activity of the refolding protein by measuring reducing sugar (glucose) hydrolyzed from laminarin. A mixture of 50 μl of refolding protein (10 μg/ml, 100 μg/ml, or 200 μg/ml) and 50 μl of 0.5% laminarin in sodium acetate buffer was incubated at 37, 40, or 50 °C for 30 min. The mixture was mixed with 100 μl of DNS reagent (1% DNS, 30% potassium sodium tartrate tetrahydrate, and 0.4 N NaOH), boiled for 5 min, cooled down to room temperature, and measured for absorbance at 540 nm using a GENESYS 10S UV-Vis spectrophotometer (Thermo Scientific). Enzyme kinetics was calculated by calibrating with standard glucose solutions.

### 2.8 Assessment of immunoreactive property of the recombinant Exo1

The recombinant Exo1 protein was assessed for immunoreactivity against the anti-*P. insidiosum* antibodies by ELISA and Western blot. ELISA was conducted by using the method of Jaturapaktrarak et al., (2020) and serum samples from 12 pythiosis patients (who had the *P. insidiosum* infection at medium-to-large size arteries of lower extremities and suffered from arterial insufficiencies, such as claudication and gangrenous ulcer) and 12 healthy blood donors (control). The presence or absence of the anti-*P. insidiosum* antibodies in each serum were confirmed by the established immunochromatographic test (ICT) (Intaramat et al., 2016). A 96-well polystyrene plate (Corning) was coated with 100 μl of the denatured Exo1 (5 μg/ml) and incubated with 200 μl of the blocking buffer [1% BSA in PBS-T (PBS with 0.1% Tween 20)]. ELISA analyzed all serum samples (1:1,500 in blocking buffer; 100 μl/well) in duplicate. The protein A/G conjugated with horseradish peroxidase (Thermo Scientific) was diluted (1:100,000) in blocking buffer and added to each well (100 μl/well). ELISA signals were developed using a Pierce TMB substrate kit (Thermo Scientific) and measured at 450-nm absorbance by an Infinite M200 PRO plate reader (Tecan). Obtained ELISA values were processed by the box-and-whisker plot. The statistical difference between the pythiosis and control sera was calculated using the single factor (one-way) ANOVA in the MS Excel program. For Western blot analysis, the Exo1-blotted membrane was probed against 3 each of the pythiosis and control sera (1:2,000 in blocking buffer) with the highest, medium, and lowest ELISA signals. The goat anti-human IgG conjugated with horseradish peroxidase (Bio-Rad) was used as the secondary antibody (1:5,000 in blocking buffer).

## 3 Results

### 3.1 Structure and post-translational modifications of Exo1

**Figure 2:**
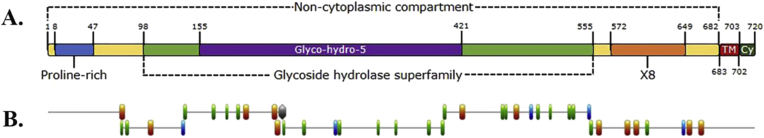
Predicted domains, cellular locations, and post-translational modifications of the Exo1 protein of *P. insidiosum*. (A) Exo1 includes a proline-rich region, a glycoside hydrolase family 5 domain (Glyco-hydro-5), an X8 domain, a transmembrane helix (TM) domain, and a cytoplasm portion (Cy). The significant portion of Exo1 (682 amino acids) accounts for extracellular (non-cytoplasmic) component, while the remaining portion (38 amino acids) appears at transmembrane and cytoplasmic locations. (B) Post-translational modifications of the Exo1 include myristoylation (labeled in orange), phosphorylation (green), *N*-linked glycosylation (blue), and ATP/GTP-binding site (gray). Numbers indicate the amino acid positions.

The ProtParam, InterPro, and ScanProsite programs predicted the functional domains and biochemical properties of the Exo1 protein (Figure 2). Exo1 was a 720-amino-acid-long acidic protein with a theoretical pI of 5.83 and a molecular weight of 82 kDa. It was predicted to be a soluble protein with the grand average of hydropathicity of -0.43. The protein contained several domains, including a proline-rich region (Prosite ID: PS50099), a glycoside hydrolase superfamily (InterPro ID: IPR017853), a glycoside hydrolase family 5 (InterPro ID: IPR001547), and an X8 domain (InterPro ID: IPR012946). The significant portion of Exo1 (682 amino acids) accounted for extracellular (non-cytoplasmic) component, while the remaining portion (38 amino acids) appeared at transmembrane and cytoplasmic locations. Many post-translational modifications were predicted in Exo1, which included 12 myristoylation sites, 22 phosphorylation sites, 5 *N*-linked glycosylation sites, and an ATP/GTP-binding motif with P-loop pattern. All functional domains and modifications resided in the extracellular portion.

**Figure 3:**
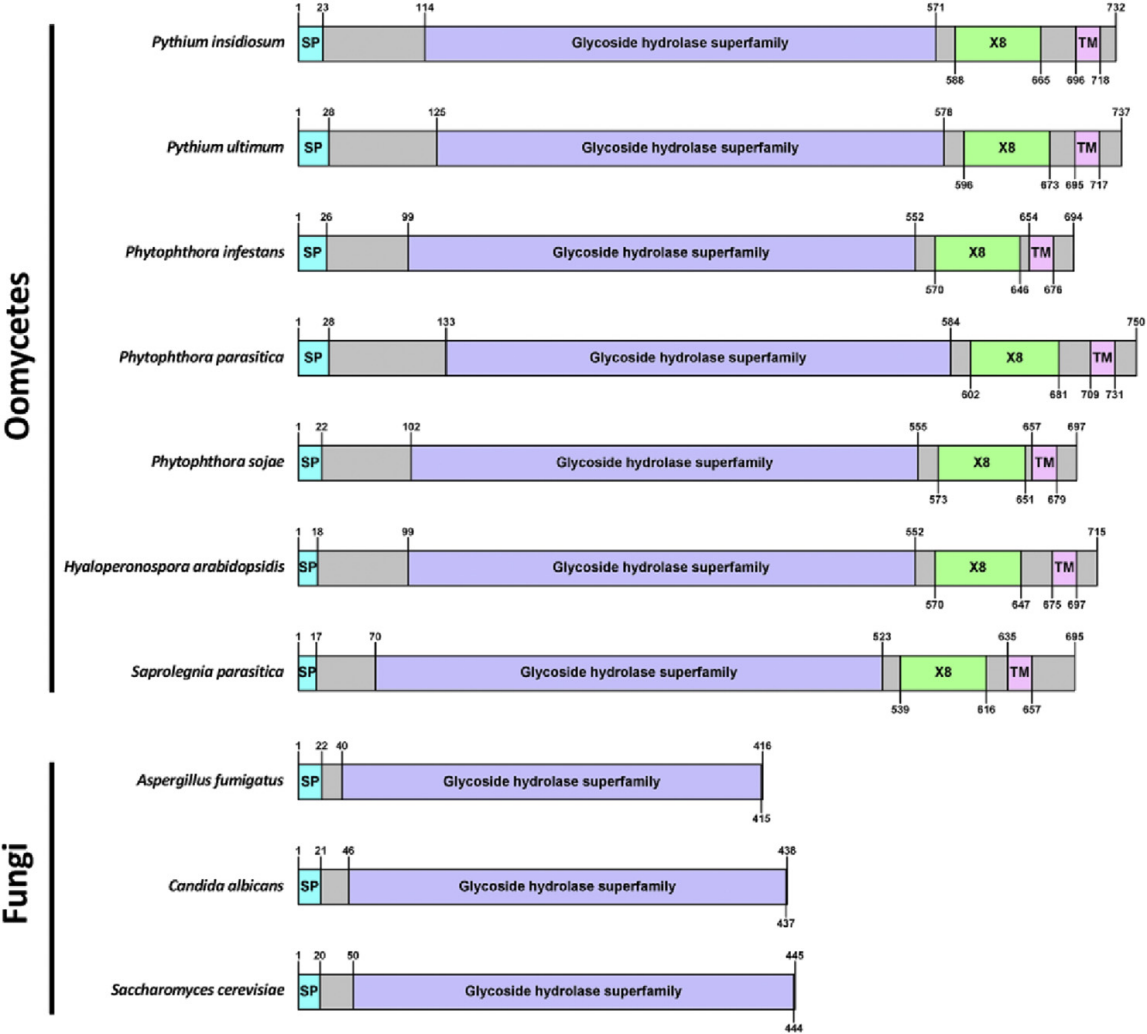
Protein architectures of exo-1,3-β-glucanases (Exo1) from *P. insidiosum*, other oomycetes, and fungi. Domain types and organizations are predicted in the Exo1 homologous proteins from *P. insidiosum* (GenBank accession: BAS04780.1), several other oomycetes [i.e., *Pythium ultimum* (FungiDB accession: PYU1_T011822), *Saprolegnia parasitica* (FungiDB accession: SPRG_13455), *Hyaloperonospora arabidopsidis* (FungiDB accession: HpaG806582), *Phytophthora infestans* (GenBank accession: XP_002903391.1), *Phytophthora parasitica* (FungiDB accession: PPTG_01939), and *Phytophthora sojae* (GenBank accession: XP_009531404.1)] and fungi [i.e., *Aspergillus fumigatus* (GenBank accession: XP_750110.1), *Candida albicans* (GenBank accession: CAA39908.1), and *Saccharomyces cerevisiae* (GenBank accession: AAB24895.1)]. ‘SP’ stands for signal peptide. Purple, green, and pink boxes show glycoside hydrolase superfamily, X8, and transmembrane (TM) domains, respectively. Numbers indicate amino acid positions.

The protein lengths and domain organizations of Exo1 homologs from oomycetes (including *P. insidiosum*) and fungi were predicted and compared, as shown in Figure 3. All oomycete Exo1 homologs (lengths: 694–750 amino acids) possess one each of signal peptide, glycoside hydrolase superfamily domain, X8 domain, and transmembrane domain. All fungal Exo1 homologs (lengths: 416–445 amino acids) contain an only signal peptide and glycoside hydrolase superfamily domain.

### 3.2 Expression, purification, validation, and functional assessment of Exo1

**Figure 4:**
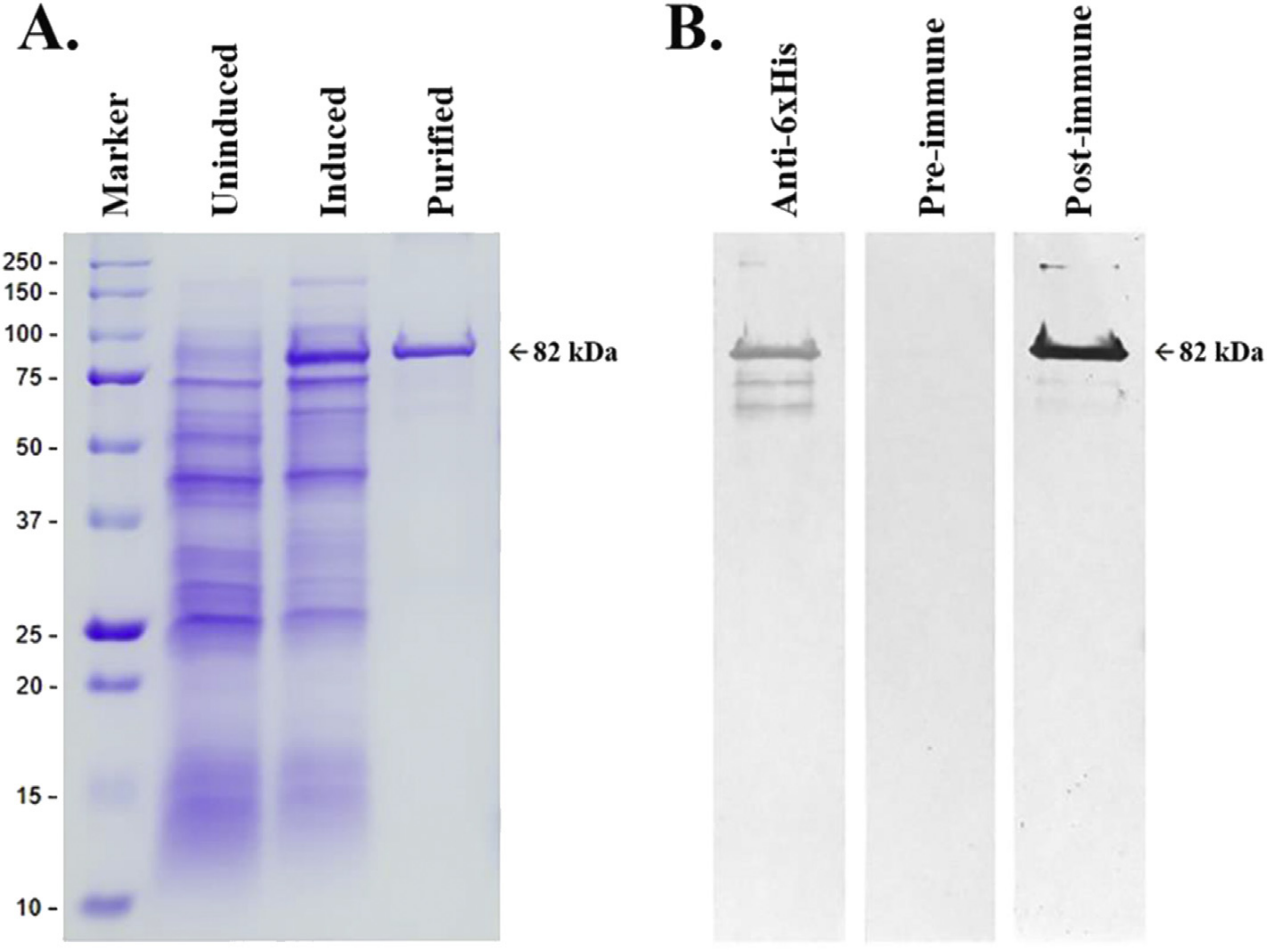
SDS-PAGE and Western blot analysis of the recombinant Exo1 protein of *P. insidiosum*. (A) SDS-PAGE of *E. coli* lysate demonstrates a prominent soluble 82-kDa band (indicated by arrow), which is compatible with the recombinant Exo1 protein, after IPTG induction (‘Induced’ lane), whereas no such band is clearly observed without the IPTG induction (‘Uninduced’ lane). Affinity column chromatography-based purification reveals the recombinant 82-kDa Exo1 protein (‘Purified’ lane). (B) Western blot analysis shows the purified recombinant 82-kDa Exo1 protein (indicated by arrow) is probed by the mouse anti-6x histidine tag monoclonal antibody (‘Anti-6xHis’ lane), and the rabbit pre-immune (‘Pre-immune’ lane) and post-immune (‘Post-immune’ lane) sera against Exo1 peptides. Protein molecular weight markers (kDa) are shown on the left. The original, unprocessed, unlabeled images of Figure 4 are available as the supplementary files SupFig4A_SDS-PAGE.jpg, SupFig4B_Anti-6xHis.tif, and SupFig4B_PrePost-immune.tif.

The *exo1* coding sequence (2,163-bp long) was codon-optimized and synthesized for constructing the expression plasmid pBT7-Exo1 (Figure 1). The *E. coli* strain BL21(DE3)pLysS was used to express the 6xHis-tagged recombinant Exo1 protein (excluding the signal peptide), which is 735 amino acids long. SDS was used to rupture the bacterial cells and solubilize the recombinant Exo1. The soluble 82-kDa Exo1 protein was observed only in the crude lysate prepared from the IPTG-induced bacterial culture, but not the uninduced culture (Figure 4A). The SDS level in the Exo1 solution was diminished by low-temperature incubation, centrifugation, and dialysis. The recombinant Exo1 was purified by affinity column chromatography in a denaturing condition using a urea-based buffer (Figure 4A). Approximately 0.5 mg of the denatured Exo1 protein was obtained from 100 ml of bacterial culture.

**Figure 5:**
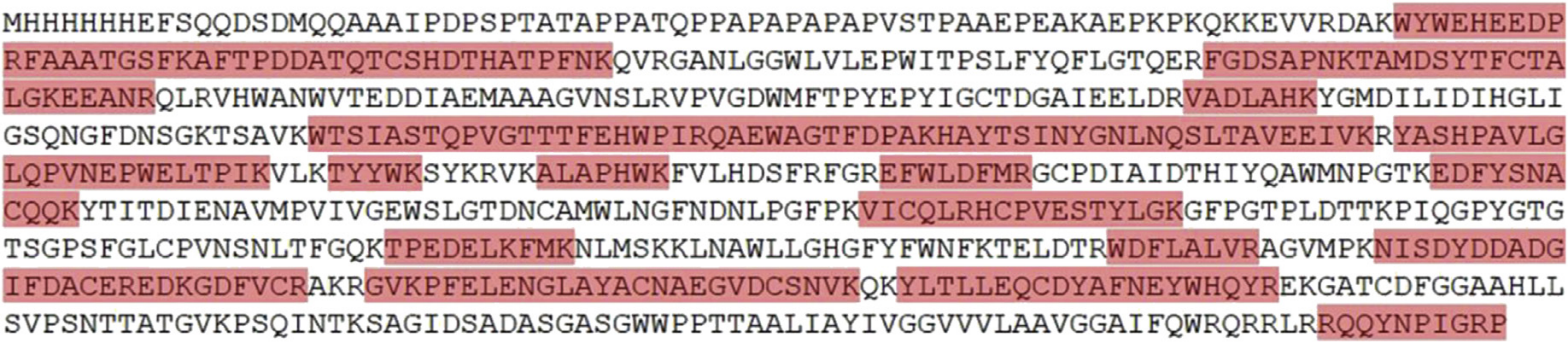
LC-MS/MS analysis of the recombinant 82-kDa Exo1 protein of *P. insidiosum*. Mass spectrometric data generated from the gel-excised 82-kDa Exo1 protein can map 302 amino acids (labeled in red) of this 735 amino acid long protein.

The identity of the purified recombinant Exo1 was confirmed by Western blot and mass spectrometric analyses. Western blot showed that the mouse anti-6xHis tag monoclonal antibody and the rabbit post-immune serum (containing anti-Exo1 peptide polyclonal antibodies), but not the rabbit pre-immune serum (Keeratijarut et al., 2015), strongly reacted the recombinant 82-kDa Exo1 protein (Figure 4B). Several faint immunoreactive bands with sizes of less than 82 kDa were observed. The recombinant 82-kDa Exo1, excised from the SDS-PAGE gel, was subject to LC-MS/MS analysis. The obtained mass spectrometric data mapped 302 amino acids (41%) of the 735 amino acid-long Exo1 protein (Figure 5).

**Figure 6:**
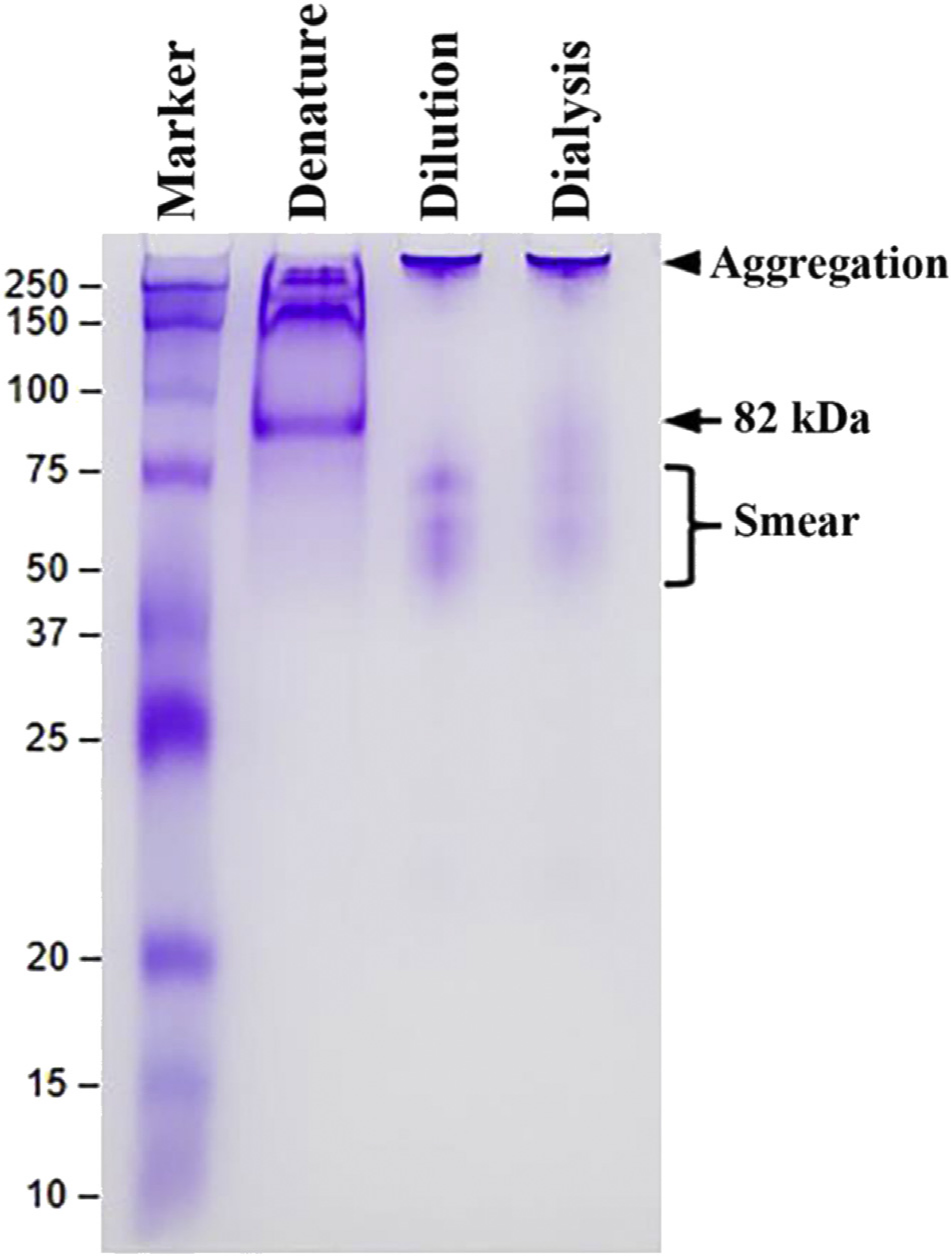
Refolding of the denatured Exo1 protein of *P. insidiosum*. A Coomassie Brilliant Blue-stained native PAGE gel shows the denatured Exo1 (‘Denature’ lane), and the Exo1 protein refolded by the dilution (‘Dilution’ lane) and step-wise dialysis (‘Dialysis’ lane) methods. The arrowhead indicates protein aggregation. The arrow locates the 82-kDa Exo1 protein. The bracket demonstrates the smeared proteins observed in the ‘Denature’ and ‘Dialysis’ lanes. Protein molecular weight markers (kDa) are shown on the left. The original, unprocessed, unlabeled image of Figure 6 is available as the supplementary file SupFig6_nativePAGE.jpg.

The denatured Exo1, solubilized in high urea concentration, underwent protein refolding processes, using the dilution and step-wise dialysis methods. A native PAGE gel showed aggregated (at the protein loading well) and smeared (from 40 to 80 kDa) bands of the Exo1 treated by each method (Figure 6). In the same native PAGE gel, the untreated denatured Exo1 protein appeared as multiple bands (misfolded fragments) ranging from 150 to 250 kDa, and as a prominent band of 82-kDa size. The agar plate enzymatic assay of the refolded Exo1 protein failed to demonstrate hydrolytic activity (no clear zone on both cellulose and laminarin-supplemented agars), as opposed to the positive controls, i.e., cellulase and lysing enzyme. A similar result was obtained from the DNS assay, in which the reducing sugar released from the hydrolysis of laminarin was undetected (data not shown).

### 3.3 Immunoreactivity of the recombinant Exo1

**Figure 7:**
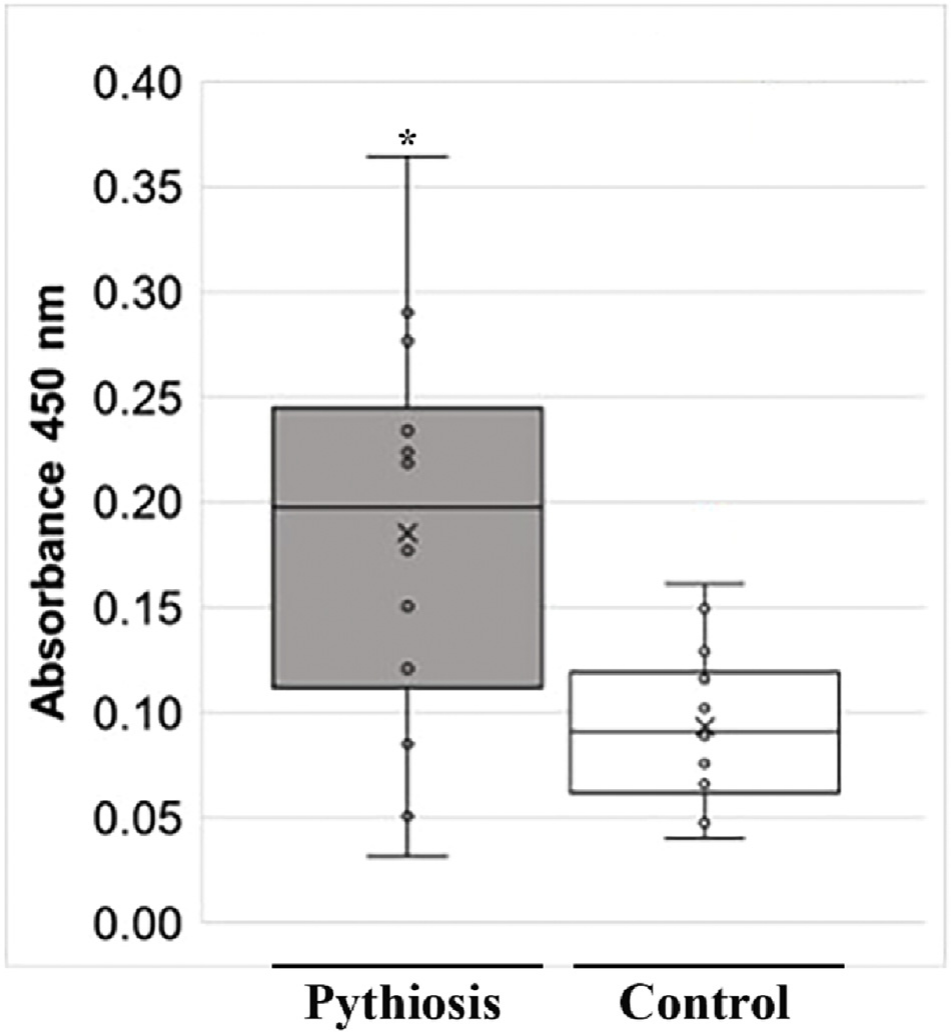
Immunoreactivity of the recombinant Exo1 protein of *P. insidiosum* by ELISA. The recombinant Exo1 protein-coated ELSIA plate is tested against 12 each of the pythiosis patient and blood donor (control) serum samples. The obtained ELISA values, measured at the 450-nm absorbance, are shown using the box-and-whisker plot. Each dot represents an ELISA value of each serum sample. The ‘x’ sign indicates the average ELISA value. The asterisk denotes the significant statistical difference between the pythiosis and control groups (*P*-value < 0.05).

**Figure 8:**
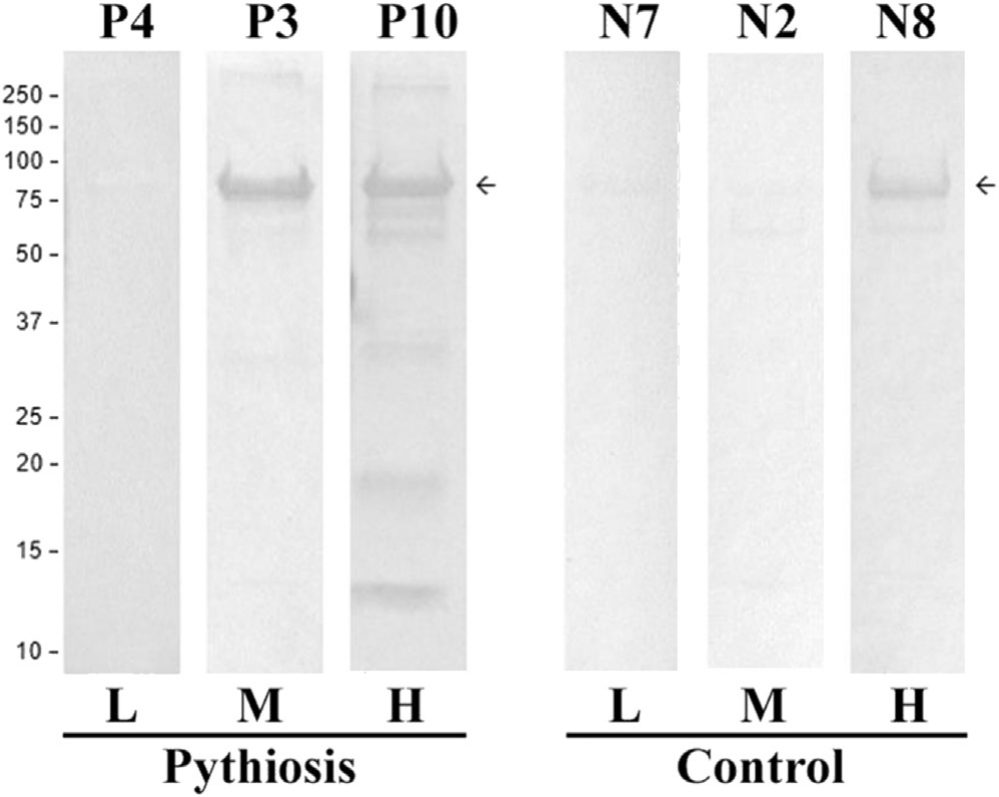
Immunoreactivity of the recombinant Exo1 protein of *P. insidiosum* by Western blot. The membrane-blotted purified recombinant Exo1 protein is tested against the serum samples with lowest (L)-, medium (M)-, and highest (H)-level ELISA values from the pythiosis patients (P4, P3, and P10, respectively) and blood donors (controls; N7, N2, and N8, respectively). The arrow indicates the 82-kDa Exo1 protein. Protein molecular weight markers (kDa) are shown on the left. The original, unprocessed, unlabeled images of Figure 8 are available as the supplementary files SupFig8_WB-Pythiosis.tif and SupFig8_WB-Control.tif.

The immunoreactivity of the recombinant Exo1 was assessed by ELISA and 24 serum samples from pythiosis patients (P1-12) and healthy blood donors (controls; N1-12) (Figure 7). The average ELISA value of the pythiosis sera (median, 0.20; mean, 0.19; SD, 0.10) was significantly higher than that of the control sera (median, 0.09; mean, 0.09; SD, 0.04) (*P*-value = 0.0079). The immunoreactivity of Exo1 was also evaluated by Western blot and the serum samples with lowest-, medium-, and highest-level ELISA values from the pythiosis patients (P4, P3, and P10, respectively) and the controls (N7, N2, and N8, respectively). The 82-kDa Exo1 band was reacted with the serum samples P10, P3, and N8, but not the serum samples P4, N2, and N7 (Figure 8).

## 4 Discussion

Bioinformatic analysis predicted the proline-rich, glycoside hydrolase family 5, and X8 domains in the exo-1,3-β-glucanase (Exo1) of *P. insidiosum* (Figure 2A). The proline-rich domain involves protein-protein interaction, signal transduction, ligand binding, regulation of biological structures, and enhancing catalytic efficiency (Ball et al., 2005; Irfan et al., 2016; Li et al., 2014; Williamson, 1994). Based on the Carbohydrate-Active Enzymes database (CAZy), the presence of glycoside hydrolase family 5 is a characteristic of some carbohydrate-metabolizing enzymes, including exo-1,3-β-glucanase, cellulase, β-glucosidase, chitosanase, and endo-1,3(4)-β-glucanase (Lombard et al., 2014). In plants, the X8 domain binds 1,3-β-glucans, such as callose and laminarin (Barral et al., 2005; Simpson et al., 2009). The recombinant Exo1 of *P. insidiosum* could possess a glycoside hydrolase activity, mediated by these predicted domains. Exo1 was also predicted to reside at the cell membrane, and 92% of its entire length, covering all domains and modifications (Figure 2), projected outward to the cell wall, which markedly consists of 1,4-β-glucan (cellulose), 1,3-β-glucan, and 1,3/1,6-β-glucan (Bowman and Free, 2006; Mélida et al., 2013). The predicted cellular location of Exo1 supported its proposed function in cell wall remodeling and hyphal growth, which are essential biological and pathogenesis-related features in other organisms (Adams, 2004; Martin et al., 2007; Sandini et al., 2007).

The protein lengths and domain organizations of oomycete and fungal Exo1 homologs are different (Figure 3). All oomycete Exo1 contain a signal peptide, 2 catalytic domains, and a transmembrane domain, suggesting that they possess carbohydrate hydrolytic activity and locate at the cell membrane. In contrast, all Exo1 of fungi are relatively shorter than that of oomycetes and contain only a signal peptide and a glycoside hydrolase domain, suggesting that the fungal enzymes are secreted to extracellular space. The differences in protein architecture and cellular location of these Exo1 homologs could be associated with the fact that the cell wall biology of oomycetes (composed of cellulose and β-glucan) and fungi (composed of chitin, chitosan, α-mannan, α-glucan, and β-glucan) are evolutionarily diverse (Kwon-Chung, 1994).

The recombinant Exo1 protein, when available, could facilitate its functional study, and also serve as a candidate for the development of a novel diagnostic or therapeutic modality for pythiosis. Our previous attempts failed to express and purify Exo1 in *E. coli* (Keeratijarut et al., 2015). Expression of a heterogenous gene in *E. coli* could lead to the formation of an inclusion body (i.e., insoluble, aggregated, and misfolded proteins) due to improper host cell environment (i.e., pH, osmolarity, redox potential, and folding mechanisms) (Carrió and Villaverde, 2002), excessive protein production (Carrió et al., 2005), and hydrophobic nature of the membrane protein (Cunningham and Deber, 2007; Seddon et al., 2004). In the current study, we codon-optimized the *exo1* gene of *P. insidiosum* to fit best the protein synthesis machinery of *E. coli*. We also used SDS (a strong anionic detergent) to rupture the bacterial cells and prevent the expressed Exo1 from aggregation, as noted by other studies (Bhuyan, 2010; Schlager et al., 2012). Although SDS solubilized the recombinant Exo1, it could interfere with the histidine and nickel affinity interaction and eventually compromise the protein purification (Schlager et al., 2012). Therefore, the SDS concentration was diminished (by low-temperature incubation, centrifugation, and dialysis) to an adequate level that did not interfere with the binding of the target protein to the column. The denaturant was changed to a high concentration of urea, a chaotropic agent that is suitable for the affinity column chromatography-based purification. As shown here, denaturing and stabilizing Exo1 with SDS- and urea-based buffers, aimed at preventing protein misfolding and aggregation, can result in the successful expression and purification of this challenging protein (Figure 4A).

The lack of enzymatic activity of Exo1 against cellulose and laminarin may occur due to the absence of proper protein folding and post-translational modifications, as the protein was expressed in the bacterium *E. coli* system. SDS- and urea-induced protein denaturation (i.e., loss of secondary and tertiary structures) could also affect the biochemical property and stability of Exo1. Our attempts in regaining the proper structure of the denatured Exo1 by both dilution and step-wise dialysis methods failed because the aggregated and smeared Exo1 protein fragments were obtained (Figure 6), and all refolding protein samples did not show any enzymatic activity. Western blot analyses using the mouse anti-6xHis tag antibodies and the rabbit anti-Exo1 peptide antibodies revealed that a small portion of the expressed Exo1 protein was degraded, and several faint immunoreactive bands were observed at sizes <82 kDa (Figure 4B).

Regarding the immunological property, Exo1 of *P. insidiosum* was reactive against the pythiosis sera tested, and the average ELISA value of the sera from the pythiosis patients was significantly higher than that from the control individuals (Figure 7). This result suggests that the patient antibodies recognize linear epitopes presented in the denatured form of Exo1. However, based on the Western blot assay (Figure 8), Exo1 was unrecognized by the pythiosis serum P4 (which had a relatively-low ELISA value) while recognized by the control serum N8 (which had a relatively-high ELISA value). The serum P4 might be derived from a pythiosis patient infected with a *P. insidiosum* strain that minimally expressed Exo1 and resulted in an undetected antibody response. The non-specific recognition of Exo1 by N8 may due to an individual (the blood donor whom the serum was derived from) exposed to an Exo1 homolog from other microorganisms and thus generated cross-reactive antibodies.

In conclusion, we successfully cloned, expressed, and purified the immunoreactive Exo1 protein of *P. insidiosum*. Exo1 was predicted to reside at the cell membrane and involve in cell wall remodeling, hyphal growth, and invasion, which are pathogenesis-related features of some fungal pathogens. The absence of enzymatic activity of the recombinant Exo1 was due to the lack of proper folding and post-translational modifications. The unrecognition and cross-reactivity of Exo1 by some pythiosis and control sera suggested that the *E. coli*-derived Exo1 was not a suitable candidate for the development of a diagnostic test. The recombinant Exo1 can be produced at an unlimited amount. Taken into account its conserve and immunoreactive feature across various *P. insidiosum* strains (Krajaejun et al., 2006a, 2006b, 2010), the recombinant Exo1 could serve as an extra protein to enhance the effectiveness of the current form of the crude extract-derived vaccine against pythiosis.

## Declarations

### Author contribution statement

Tiwa Rotchanapreeda: Conceived and designed the experiments; Performed the experiments; Analyzed and interpreted the data; Wrote the paper.

Yothin Kumsang, Pattarana Sae-Chew, Thidarat Rujirawat, Wanta Yingyong, Penpan Payattikul: Performed the experiments.

Tassanee Lohnoo: Performed the experiments; Contributed reagents, materials, analysis tools or data.

Onrapak Reamtong: Performed the experiments; Analyzed and interpreted the data; Contributed reagents, materials, analysis tools or data.

Theerapong Krajaejun: Conceived and designed the experiments; Analyzed and interpreted the data; Contributed reagents, materials, analysis tools or data; Wrote the paper.

### Funding statement

This work was supported by Postdoctoral Fellowship Grant from 10.13039/501100004156Mahidol University, Thailand (Grant number: MU-PD_2020_7 [T. Rotchanapreeda]); 10.13039/501100004396Thailand Research Fund, Thailand (Grant number: RSA6280092 [T. Krajaejun]); and 10.13039/501100010804Faculty of Medicine, Ramathibodi Hospital, 10.13039/501100004156Mahidol University, Thailand (Grant number: CF_61007 [T. Krajaejun]).

### Competing interest statement

The authors declare no conflict of interest.

### Additional information

No additional information is available for this paper.

## Figures and Tables

**SupFig8_WB-Control:**
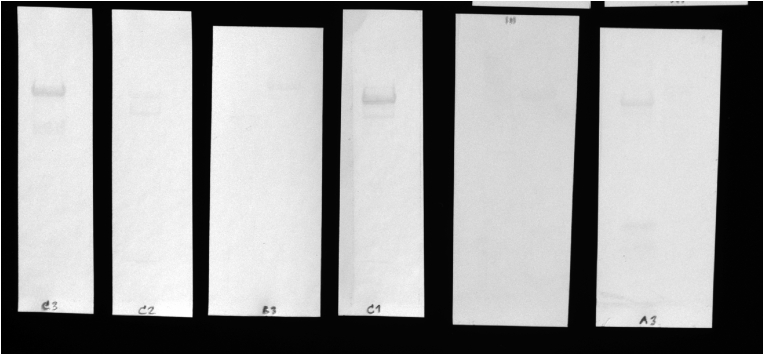


**SupFig4B_Anti-6xHis:**
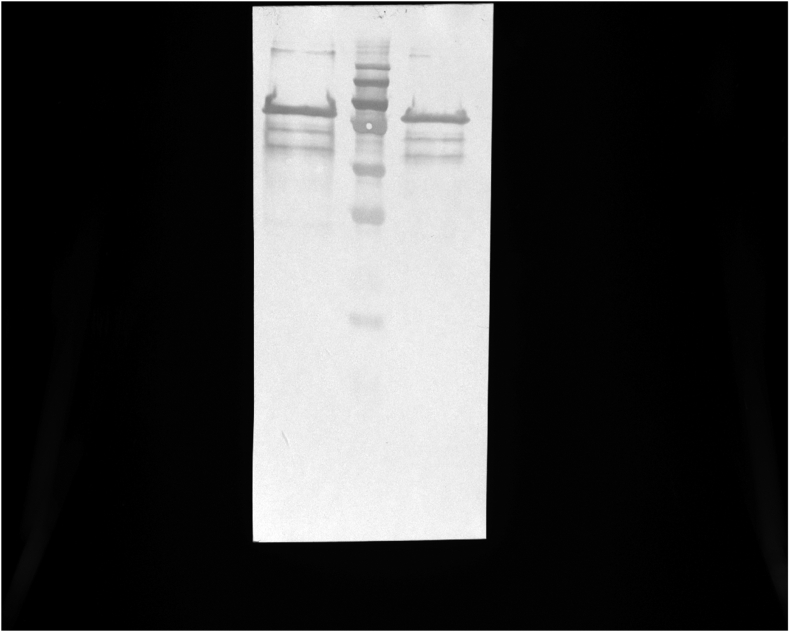
2

**SupFig4B_PrePost-immune:**
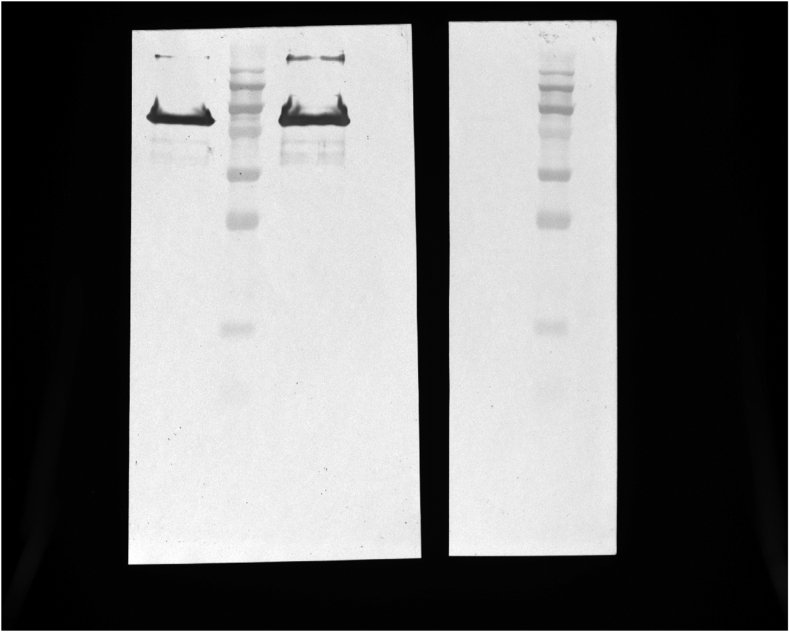
3

**SupFig6_nativePAGE:**
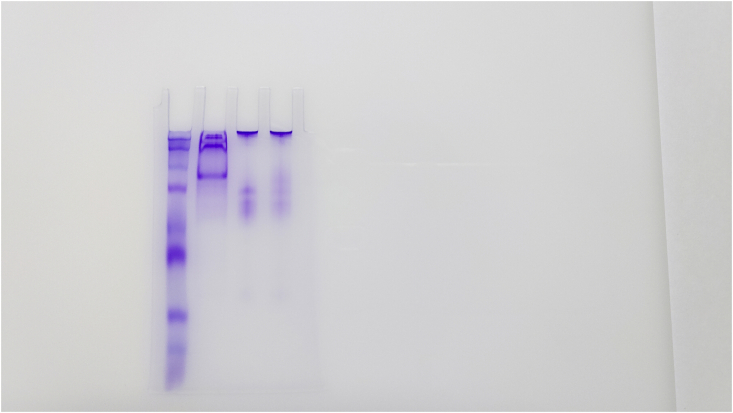
4

**SupFig8_WB-Pythiosis:**
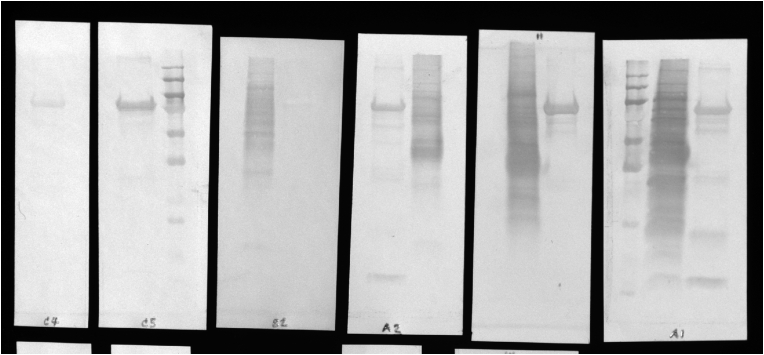
5

**SupFig4A_SDS-PAGE:**
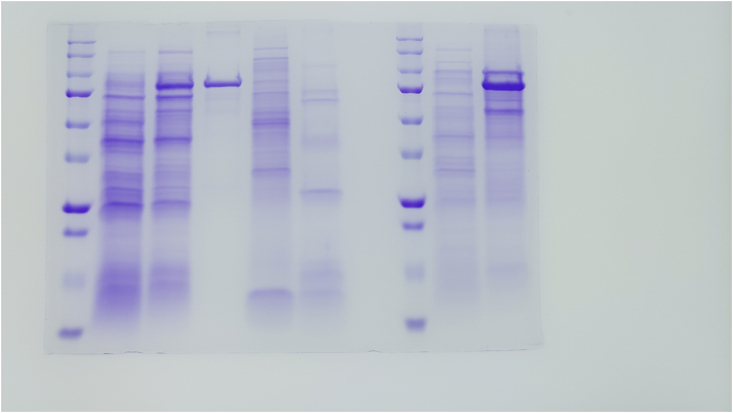
6
